# 2019 Coronavirus Disease Applications Made During an Outbreak in a Chemotherapy Center: The Case of Turkey

**DOI:** 10.1017/dmp.2020.188

**Published:** 2020-06-05

**Authors:** Yasemin Özyer

**Affiliations:** Sinop University, Durağan Vocational School, Medical Services and Techniques Department

**Keywords:** 2019-nCoV, chemotherapy, COVID-19, health care

In December 2019, the pneumonia epidemic that occurred due to an unknown reason in Wuhan, China, had spread throughout the whole country.^1^ This disease was originally named the *novel coronavirus (2019-nCoV)*, then the World Health Organization (WHO) changed the name of this epidemic disease officially to *coronavirus disease 2019 (COVID-19)* on February 11, 2020. After this date, 37 590 cases were detected worldwide.^2^ To date, COVID-19 has become a global threat, affecting more than 28 countries.^1^ It is stated that the number of cases reported from China is 70 635 and the remaining 794 cases are from 24 different countries.^3^ Turkey had the first person-to-person infection on March 10, 2020. As of April 18, 2020, in Turkey, with 82 329 confirmed cases, 1890 deaths, and 10 453 patient-recovery cases so far, numbers have been changing hours by hours.^4^ All public hospitals in the world and in our country face a difficult battle in this epidemic process. The COVID-19 outbreak poses a challenge for the treatment of cancer patients and patient safety. Timely and comprehensive planning is very important for maintaining health and treatment services. The chemotherapy unit should continue to operate at normal capacity to prevent delays in cancer treatment. In this process, especially, cancer patients who receive chemotherapy treatment become more vulnerable to the virus, their immune system is weakened, and they are more susceptible to infections. Therefore, when deciding on the point of continuing treatment, the benefit/loss ratio should be determined for the patient, and if the net benefit is high, chemotherapy treatment should be continued. In the patient’s asymptomatic process, discontinuation of the treatment or stopping the treatment is a more correct decision; when patients are infected with the virus that causes COVID-19, patients suffer much more than they benefit on adjuvant therapy and they are at risk of death.^5^

In this study, we aimed to present solutions for health care professionals, nurses, and patients working in a hospital’s oncology department, chemotherapy unit.

## WHAT IS DONE IN DISEASE PROCESS MANAGEMENT

The primary goal is to determine the priority of treatments, restructure the services provided during the COVID-19 outbreak, and, most importantly, ensure that patients are treated with the most benefit and least harm during this process.^5^

Because of the immunosuppressive treatment of cancer patients, their immune systems are less effective and the patients become susceptible to infection. In this respect, patients should continue their routine chemotherapy treatments in the chemotherapy unit according to the condition of their immune system. Ensuring the safety of health care professionals and patients, determining infected individuals, and preventing contamination to other individuals gain importance in managing the process.

During the COVID-19 outbreak, a strict and safe triage procedure was applied to evaluate the treatment of cancer patients, and their urgency and necessity of hospitalization. Patients and employees were encouraged to use the hydroalcoholic hand disinfectant located in the chemotherapy unit and its entrance. The training was given on the importance of hand hygiene and personal hygiene. In order to minimize occupational exposure to the virus and reduce the risk of contamination, adequate personal protective equipment (PPE) was provided for health care workers and infection prevention and control measures in health care facilities were implemented meticulously. With the increased use of PPE, protective measures were taken as hospital management for deficiencies in protective equipment (such as tracking the number of materials, preventing unnecessary use of materials). For patients with a positive COVID-19 test results, COVID-19 isolation procedures were followed according to the hospital’s infection control committee instructions, and patients were quarantined to continue their treatment. To prevent potential confusion and infection exposure, patients were provided with same day blood analysis and chemotherapy treatment and the social distance rule was practiced. The implementation of flexible working hours, compliance with the social distance rule, and reduction of the risk of infection are among the measures taken to facilitate the provision of safe services of health workers. The number of employees was rearranged according to the number of patients. In cases of staff shortage, the current situation was reported to the hospital management, and staff support was provided to prevent interruption of ongoing treatments. The importance of coming to the chemotherapy unit as little as possible and delaying chemotherapy treatments for patients with better prognosis and non-urgent patients has been emphasized. Patient visits are restricted.

## DISCUSSION

The COVID-19 epidemic in the world and Turkey continues to increase. Studies show that patients with cancer are at higher risk for COVID-19 disease than non-cancer patients.^5^ Chemotherapy centers in hospitals, which first encountered an emergency, adapted to the event very quickly and continued to provide routine treatment to their patients. Planning flexible working hours for the medical staff, applying the social distance rule, reducing outpatient visits, and rearranging the chemotherapy treatment according to the patient’s prognosis are among the measures taken for COVID-19 in order to reduce the risk of infection and to prevent transmission. Although PPE is available for employees, in case of material deficiency, protection methods should be applied for all PPEs.

Preparing drugs with the robotic infusion system, calling patients using the appointment system without making them wait when the drug is ready, performing the analysis and treatments on a single day, and giving the chemotherapy drugs to the patient as an oral drug instead of parenteral administration are among the different applications for patients and employees, in comparison with other institutions.

In our study, we aimed to briefly discuss what needs to be done to ensure the continuity of treatment while protecting patients, health care workers, and the general population, and providing recommendations during emergencies. What to do is integrated according to WHO recommendations and health authorities’ guidelines. This study focuses mainly on how health care professionals working in chemotherapy centers can deal with symptomatic, suspected, or confirmed COVID-19 patients and what measures to take.
